# Quantitative Structure-Activity Relationship (QSAR) Studies on the Toxic Effects of Nitroaromatic Compounds (NACs): A Systematic Review

**DOI:** 10.3390/ijms22168557

**Published:** 2021-08-09

**Authors:** Tao Huang, Guohui Sun, Lijiao Zhao, Na Zhang, Rugang Zhong, Yongzhen Peng

**Affiliations:** 1 Key Laboratory of Environmental and Viral Oncology, College of Life Science and Chemistry, Faculty of Environment and Life, Beijing University of Technology, Beijing 100124, China; huangtaovincent@gmail.com (T.H.); zhaolijiao@bjut.edu.cn (L.Z.); nanatonglei@bjut.edu.cn (N.Z.); lifesci@bjut.edu.cn (R.Z.); 2 National Engineering Laboratory for Advanced Municipal Wastewater Treatment and Reuse Technology, College of Environmental and Chemical Engineering, Faculty of Environment and Life, Beijing University of Technology, Beijing 100124, China; pyz@bjut.edu.cn

**Keywords:** nitroaromatic compounds, in silico modeling, QSAR, animal testing, toxicity

## Abstract

Nitroaromatic compounds (NACs) are ubiquitous in the environment due to their extensive industrial applications. The recalcitrance of NACs causes their arduous degradation, subsequently bringing about potential threats to human health and environmental safety. The problem of how to effectively predict the toxicity of NACs has drawn public concern over time. Quantitative structure–activity relationship (QSAR) is introduced as a cost-effective tool to quantitatively predict the toxicity of toxicants. Both OECD (Organization for Economic Co-operation and Development) and REACH (Registration, Evaluation and Authorization of Chemicals) legislation have promoted the use of QSAR as it can significantly reduce living animal testing. Although numerous QSAR studies have been conducted to evaluate the toxicity of NACs, systematic reviews related to the QSAR modeling of NACs toxicity are less reported. The purpose of this review is to provide a thorough summary of recent QSAR studies on the toxic effects of NACs according to the corresponding classes of toxic response endpoints.

## 1. Introduction

Nitroaromatic compounds (NACs) are a type of aromatic compound with at least one nitro group (-NO_2_) located at the benzene ring. They comprehensively exist in the atmospheric, aquatic, and terrestrial environment, as well as human foods. The nitro group on benzene ring delocalizes π-electrons of the ring, satisfying the deficiency of its own charge [1]. This unique structure enables NACs to exhibit various applications. For example, most explosive materials manufactured in the last century were NACs, such as 2,4,6-trinitrotoluene (TNT), 1,3,5-trinitrobezene (TNB), tetryl nitramine, trinitrophenols, hexanitrobezene, etc. [2,3]. In the dye industry and pharmaceutical factories, 2-nitrophenol derivatives are widely used. 2,4-Dinitrotoluenes (DNTs) are used in organic synthesis industries such as polyurethane, dyes, medicine, and rubber. Dinitrophenols (DNPs) have been used in herbicides and pesticides [4]. Table 1 lists the names, sources, and applications of NACs, and their toxic effects observed in different organisms. The main source of NACs in the environment is artificial manufacture (industrial emission, automobile exhaust and domestic sewage) due to the comprehensive application in industry. Apart from anthropogenic activities, some NACs can be produced by certain microbes, parasites or plants as secondary metabolites or pheromones [5]. The psychrotolerant, gram-negative bacterium *Salegentibacter* sp. strain T436, found in the Arctic, can produce 21 monocyclic NACs as secondary metabolites. The fermentation can be optimized to improve their antimicrobial and cytotoxic ability, of which 2-nitro-4-(2′-nitroethenyl)phenol is the most potent one [6,7]. The electron-withdrawing nitro groups cause NACs to be recalcitrant for degradation [8]. Furthermore, the degradation of NACs is not sustainable and efficacious enough, directly leading to their persistent accumulation in the environment. Hence, the contamination of NACs poses a serious threat to the ecological environment and human health.

NACs exhibit various toxic effects towards living organisms, such as mutagenicity, carcinogenicity, liver damage, jaundice, skin irritation, etc. It is reported that nitro polycyclic aromatic hydrocarbons (NPAHs) can be assimilated by plants and accumulated in the food chain, posing a potential danger to human [17]. Certain NACs like arylhydroxylamines, arylamines, azo, and azoxy compounds even display higher toxicity than their parent NACs [18]. The exhaust of diesel includes NPAHs such as 6-nitrochrysene, 1-nitropyrene, 1,6-dinitropyrene, etc., which are tested to be carcinogenic and mutagenic to mammals and human beings [19]. US Environmental Protection Agency (EPA) already listed them as priority pollutants [19,20]. In 2007, Germany identified more than 10 kinds of nitrophenols and nitrobenzoic acids in ground water and leachate; adjacent residents were found to show different symptoms like nausea, vomiting, headache, etc. [21]. Interestingly, a recent study concluded that 3-trifluoromethyl-4-nitrophenol (TFM) destroyed the balance of ATP supply and demand in trout [22]. TNT (including its metabolites) and DNT are manifested to impair the reproduction of crickets and salamander as well [23,24].

In general, there are three paths (physical, chemical and biological methods) to remove NACs in the environment. Physical methods involve absorption, adsorption, extraction, ultrafiltration, photo-oxidation, and volatilization. Among them, adsorption is employed most extensively [5]. Chemical methods include hydrolysis and advanced oxidation processes (AOPs). AOPs utilize oxidants like Fenton reagent, H_2_O_2_, and metal as photocatalysts to oxidize NACs. In this case, the stability of the aromatic ring is destroyed by radicals generated from oxidants, thus leading NACs to be decomposed into smaller molecules [25]. Biodegradation refers to using microorganisms to decompose NACs, typically including aerobic or anaerobic approach. Even if particular NACs are inclined to aerobic degradation, a number of them are recalcitrant to be oxidized. NACs with high electronegativity are amenable to be reduced first. Besides, it is difficult to decompose NACs completely solely by bacteria, and microbes differ in their ability to degrade NACs. Therefore, the selection of appropriate microorganisms is of vital importance [26]. The major defect of physical methods is that adsorbents usually adsorb organic compounds indiscriminately and such pollutants require further treatment to detoxify. AOPs are costly and microorganisms are inefficient in high concentration scale [5]. Consequently, physico-chemical and biological methods are integrated to remove NACs in modern industry. In this case, NACs may be further detoxified to meet the environmental standard. However, the economic costs are very high and non-negligible. To avoid these NACs with high toxicity entering the environment, assessing various toxic effects of NACs in advance is necessary. As there is a massive number of NACs, it is impossible to evaluate all the toxic response endpoints through experiments in vitro or in vivo. Consequently, in silico predictive modeling techniques are introduced to supplant these experiments since they can reduce the number of animal testing, satisfy 3R rules (reduction, replacement, refinement), and rapidly predict the toxicity [27].

QSAR means quantitative structure–activity relationship, which is a process that quantitatively bridges molecular descriptors and biological activities. Molecular descriptor is the “fingerprint” of one molecule on the micro level, including theoretical and experimental parameters such as atom numbers; chemical bonds numbers; MLI (molecular connectivity index); ionization constant, pKa; electric dipole moment *μ*; MR (molecular molar refractive index), logP (lipophilic parameter), etc. With the aid of integrated modeling softwares and well-built mathematical models, the biological properties of specified functional groups can be analyzed and further utilized for chemical design to enhance or decrease the corresponding response endpoints. Common mathematical modeling algorithms include: Multiple Linear Regression (MLR), Genetic Algorithm (GA), Principal Component Analysis (PCA), Partial Least-square method (PLS), Artificial Neural Network (ANN), and Support Vector Machine (SVM). MLR, PCA, and PLS are favorable to process linear relationships, while GA, ANN, and SVM deal with non-linear relationships better [28,29]. Typically, the selection of molecular descriptors determines the quality of models. More descriptors are not meant to be more precise, on the contrary, it may result in overfitting. Accordingly, the selected molecular descriptors must be mutually independent or less correlated.

The results from QSAR are necessary to be verified and evaluated through statistical approaches, which involve internal and external validation metrics. Internal validation commonly employs leave-one-out cross validation (LOO), leave-more-out (LMO) and Y-scrambling method (also called Y-randomization). *R*^2^ is the determination coefficient of the model, which indicates its fitting ability. *Q*^2^ is the cross-validation coefficient of the model, which indicates its robustness and stability. The classical formulations of *R*^2^ and *Q*^2^ are listed as Equations (1) and (2).

(1) $$R^{2}=1-\frac{\sum {\left(y_{i}-{\hat{y}}_{i}\right)}^{2}}{\sum {\left(y_{i}-\bar{y}\right)}^{2}}=1-\frac{R\mathrm{SS}}{TSS}$$

*Y_i_* is the observed values of the response; $\bar{y}$ is the average of all the observed values; ${\hat{y}}_{i}$ is the calculated value; *RSS* represents the residual sum of squares; and *TSS* represents the total sum of squares.

(2) $$Q^{2}=1-\frac{\sum {\left(y_{i}-\hat{y_{i}}\right)}^{2}}{\sum {\left(y_{i}-\bar{y}\right)}^{2}}=1-\frac{PRE\mathrm{SS}}{TSS}$$

*Y_i_* is the observed values of the response; $\bar{y}$ is the average of all the observed values; ${\hat{y}}_{i}$ is the calculated value for each object apart from the training set; and *PRESS* means the predicted residual sum of squares.

The performance of one model is acceptable when it satisfies *R*^2^ > 0.6 (for training and test sets) and *Q*^2^ > 0.5 (for training set) [30]. A good outcome of internal validation is the prerequisite of external validation. In fact, the actual predictive capability of a QSAR model can only be assessed by datasets that are never used in model development [31,32,33]. Test set, collected outside modeling data, is applied to verify the predictive veracity of a QSAR model. Generally, the ratio of a training set to test set is approximately set as 3:1. Notably, in general, the number of descriptors in the model equation should be less than one fifth of the compounds in the training set [34]. Extra external datasets will further validate the predictive performance of a QSAR model. As of now, QSAR has been modified and ameliorated relentlessly with the development of deep learning and artificial intelligence, which guarantees the predictive ability to keep pace with the modern scientific field. Therefore, the pharmaceutical industry commonly uses QSAR methodology to design new drugs, thereby avoiding the introduction of specified toxic substructures. According to the results obtained from QSAR, the toxicity of new chemicals can be predicted, laying a solid foundation on their toxicity classification and risk assessment. Further, compared with traditional living animal testing or in vitro experiments, QSAR tremendously saves cost, time, and human resources. According to Registration, Evaluation, Authorization and Restriction of Chemicals (REACH) regulation in EU, QSAR has been promoted to apply in multiple fields since 2006 [35]. In particular, the principles for QSAR validation recommended by OECD (Organization for Economic Co-operation and Development) for regulatory purpose include: (1) a defined endpoint; (2) an unambiguous algorithm; (3) a defined domain of applicability; (4) appropriate measures of goodness-of fit, robustness, and predictivity; and (5) a mechanistic interpretation, if possible [36]. The prevalence of QSAR minimizes the use of living animals in the experiments of acute systemic toxicity and toxicokinetics [37]. 

Till today, a number of studies related to the QSAR modeling or applications for NACs toxicity have been reported. However, the information of QSAR studies on the toxic effects of NACs have not been systematically documented, compared, and reviewed. It is of vital importance to elucidate the toxic mechanism of NACs and perform the rapid toxicity prediction for developing greener and safer chemicals. In this review, we aim to summarize recent advances in QSAR studies of NACs according to the corresponding classes of toxic response endpoints.

## 2. QSAR Studies on Toxic Effects of NACs

### 2.1. Aquatic Toxicity

#### 2.1.1. QSAR Studies on Aquatic Crustaceans

QSAR studies have been performed to quantitatively evaluate the ecotoxicity of pesticides to aquatic crustaceans, such as *Daphnia magna*. According to the mode of action (MOA), most chemicals are classified as inert (baseline), polar, reactive, and specifically acting compounds [38,39,40]. It was concluded that the toxicity of inert chemicals exhibits a linear relationship with hydrophobicity, which usually measured by octanol/water partition coefficient (*LogK_ow_*). In the study performed by Wang et al., a total of 24 molecular descriptors including both physico-chemical properties and quantum chemical parameters were used to develop QSAR models [41]. The modeling dataset comprised 57 pesticides (most of them are NACs, such as Fenitrothion, Parathion, etc.); the result rendered that seven descriptors exhibited prominent correlation to the toxicity, namely, polar surface area (PSA), fraction of ionization ($F^{+}$), heat of formation ($H_{f}$), polarity (S), hydrogen bonding basicity (B), molecular volume (V), and Cosmo volume (CV). The validation of all QSAR models presented decent results with *R*^2^ > 0.8 and *Q*^2^ > 0.6. The increase of hydrophobicity contributed to an increase of the toxicity to *D. magna*. Chemical ionization can enhance the toxicity to *D. magna* as well because ionized compounds generally exhibit strong capability of interacting with biological macromolecules. Other descriptors that represent chemical stability and the strength of interaction with biological macromolecules also affect the toxicity. Molecules with strong electronegative substructures like O or N atom may show less toxicity by enhancing their overall polarity [41]. 

#### 2.1.2. QSAR Studies on Algaes

Among early QSAR studies, Zhao et al., constructed QSAR models to predict the NACs toxicity on *Scenedesmus obliguus* [42]. The toxicity of 26 NACs was demonstrated in modeling, which solely adopted internal validation (without external validation) and showed *R*^2^ = 0.86. As a corollary, descriptors such as halfwave reduction potential (*E*_12_), bioconcentration factor (BCF), the highest occupied molecular orbital energy (*E*_*HOMO*_), the lowest unoccupied molecular orbital energy (*E_LUMO_*), and octanol/water partition coefficient (*K_OW_*) were determined as algae toxicity indicators. In another study, QSAR models established by Schmitt et al., exhibited reliable results in predicting the *Scenedesmus vacuolatus* toxicity of NACs [43]. Sixteen quantum chemistry descriptors along with *K*_ow_ participated in modeling, yielding an excellent *R*^2^ = 0.9. Importantly, the prediction can be further improved by adding another descriptor maximum net atomic charge at the nitro nitrogen (*q*_nitro-N_). As a result, parameters such as *E*_HUMO_, *E_LUMO_*, *K_OW_*, and distribution coefficient proved to be highly relevant to the algae toxicity of NACs. 

In a QSAR study performed by Tugcu and co-workers, the toxicity of nitrophenols on *Chlorella vulgaris* was predicted and strictly evaluated according to OECD principles for QSAR validation [44]. The QSAR models were constructed by MLR and Ordinary Least Square (OLS) method using QSARINS software developed by Gramatica et al., [34]. A series of descriptors such as constitutional index, topological index, connectivity index, ETA index, functional group counts, etc. were totally involved in the model calculation. Individual chemical toxicity to algae is characterized by the average growth rate, which can be expressed as the half maximal inhibitory concentration (IC_50_). The results showed a sound validation both internally and externally. For training sets, $R^{2}$ > 0.6, $Q_{LOO}^{2}$ > 0.6, *RMSE* < 0.45; and for test sets, $Q_{F1}^{2}$, $Q_{F2}^{2}$ and $Q_{F3}^{2}$ > 0.70, $r_{m}^{2}$ > 0.65 and concordance correlation coefficient (*CCC*) > 0.85. Further, the predictive quality was assessed by mean absolute error (*MAE*)-based criteria proposed by Roy et al. [45]; model quality derived from Equation (3) was “good”. Considering the comprehensive performance, Equation (3) was selected to be further validated using two true external sets. As a result, the observed and predicted values of the true external chemicals were close to each other. Moreover, more than 94% chemicals in these two true external sets fell within the applicability domain (AD) of the model defined by Equation (3).

(3) $$pToxicity=0.20\left(\pm 0.17\right)LogP-2.75\left(\pm 1.33\right)Hardness+12.70\left(\pm 6.18\right)$$

According to previous studies, the octanol-water partition coefficient (*LogP*/*K_OW_*/*logK_OW_*) is an indispensable factor in chemical toxicity [1,4,24,31,41,44,46,47,48]. In some cases, though, the determinant factors of toxicity need to be replenished to account for specific phenomena like ionization [49]. In sum, descriptors like hydrophobicity, harness, and electrophilicity as key factors to algae toxicity are commonly identified from the mechanistic analysis of QSAR models.

#### 2.1.3. QSAR Studies on Aquatic Interspecies Toxicity

Interestingly, Tugcu et al., also developed Quantitatively Toxicity-Toxicity Relationship (QTTR) models to predict interspecies toxicity [44,50]. The toxicity order for dinitrophenols to *C. Vulgaris* is *para*-dinitrophenols > *meta*-dinitrophenols > *ortho*-dinitrophenols, which is identical in *T. pyriformis*. Also, the relationship among fish, bacteria, algae, and *Daphinia* toxicities had been proven in previous studies [51,52]. Ciliate–algae and algae–algae interspecies toxicity correlation were calculated through QTTR modeling. The results showed *R*^2^*_train_* = 0.75, *Q*^2^*_LOO_* = 0.72, *RMSE_train_* = 0.32, *R*^2^*_test_* = 0.82, *RMSE_test_* = 0.28; *R*^2^*_train_* = 0.93, *Q*^2^*_LOO_* = 0.91, *RMSE_tr_* = 0.18, *R*^2^*_test_* = 0.83, *RMSE_test_* = 0.27, respectively. The high values of *R*^2^*_test_* indicated a strong toxicity interrelationship within aquatic species. These models may give us insight to predict aquatic toxicity to various species through limited data [44,49].

### 2.2. Acute Toxicity

#### 2.2.1. QSAR Studies on Fish and Algae

QSAR studies on Carp acute toxicity (*LC_50_*) were established using six descriptors, namely, the first order valence molecular connectivity index (*X_v_*), the molecular shape index (*K_α_*), the sum of substituent constant (*Σσ*−), *E_LUMO_*, *logP*, and indicator variable (*I*) [53]. Nineteen NACs were involved in modeling and the best prediction was obtained according to Equation (4), inferring a close relationship in electrophilicity and toxicity of dinitro compounds as *I* (indicator variable) were considered as electron descriptors.

(4) $$-LogLC_{50}=3.522+0.926\Sigma \sigma -+0.173I\;\;\left(N=19,R=0.907,\;S=0.271\right)$$

In Yan and co-workers’ study, three descriptors *K_OW_*, *E_LUMO_*, and $Q_{NO_{2}}$ were employed to establish the QSAR models of acute toxicity (EC_50_) of algae (*Scenedesmus obliguus*) [54]. Twenty five NACs were divided into two groups: mononitro and dinitro aromatic compounds. For mononitro aromatic compounds, the authors presented a good prediction generated from *K_OW_*, which showed *R* = 0.904, indicating a strong connection between hydrophobicity and toxicity. For dinitro aromatic compounds, however, the conclusion was different due to their high electrophilic nature. Thus, the authors selected *E_LUMO_* and $Q_{NO_{2}}$ to predict dinitro aromatic compounds toxicity and showed a reasonable result: *R* = 0.926, *SE* = 0.206. Different toxic mechanisms result in different key parameters in QSAR models, thus presenting inevitable challenges to researchers. The toxicity of mononitro aromatic compounds commonly can be expressed as the narcotic toxicity, which concentrates on the penetration of the cell membrane.

#### 2.2.2. QSAR Studies on Rodents

For rodent acute toxicity, lots of QSAR models have been constructed by using rat or mouse as experimental objects [47,48,55,56,57,58]. QSAR models based on the simple representation of molecular structure (SiRMS) method were constructed by Kuz’min et al., to predict the rat oral acute toxicity (LD_50_) of 28 NACs [59]. In this study, both 1D and 2D descriptors-based models generated decent prediction: *R*^2^ = 0.96~0.98; *Q*^2^ = 0.84~0.93; *R*^2^*_test_* = 0.89~0.92. The results showed that hydrophobicity, electrostatic, and Van der Waals interactions contributed to the toxicity. What is more, the addition of fluorine and hydroxyl groups in NACs increases the toxicity, while the insertion of methyl groups decreases the toxicity in most cases. Importantly, the toxicity of substituents is non-additive. The insertion of chlorine in *ortho*-position to the nitro group apparently enhances the toxicity while the second chlorine insertion in *para*-position significantly reduces the toxicity. Furthermore, recently, Hao et al., performed the QSTR modeling of the rat acute oral toxicity of NACs and constructed an interspecies QTTR models between rat and mouse [55]. Seven simple 2D descriptors were utilized to achieve good prediction ($Q_{LOO}^{2}$ = 0.7003, $R_{adj}^{2}$ = 0.7292, $RMSE_{train}$ = 0.5628; $Q_{F1}^{2}$ = 0.7458, $Q_{F2}^{2}$ = 0.7340, $Q_{F3}^{2}$ = 0.7993, $R_{test}^{2}$ = 0.7593, $RMSE_{test}$ = 0.5024). Specifically, the van der Waals surface area, the presence of C-F at topological distance 6, and high frequency of C-N at topological distance 9 directly affect the acute oral toxicity of NACs to rat. The rat–mouse and mouse–rat interspecies QTTR models also had excellent internal and external predictive performance and thus can be used for interspecies data gap filling [55]. More importantly, the models were used for a true external set containing hundreds of NACs without experimental values; the AD analysis showed that more than 90% compounds fell within the AD of the models. To better comprehend the mechanism of NACs acute toxicity in mammals, the authors also developed QSAR models by using lipophilic descriptor *logP* as the only descriptor, showing a poor correlation between the rat acute toxicity of NACs and *logP* [55]. Unlike aquatic toxicity, *logP* (*logK_OW_*) has little impact on the rat acute toxicity of NACs. One possible explanation is that the toxic mechanisms of terrestrial mammals are far more intricate than aquatic toxicity since NACs’ reactions with organisms in vivo may play more important roles than molecular diffusion.

**Figure 1:**
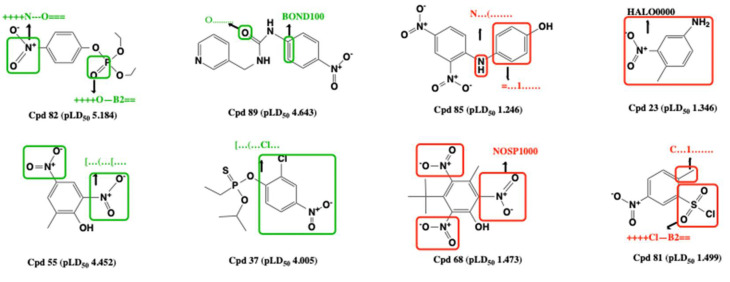
Molecular fragments for toxicity promotors (green) and inhibitors (red).

Using physico-chemical and quantum chemistry descriptors sometimes are not enough to obtain a satisfactory toxicity prediction, thus Mondal et al., established QSAR models by using specific substructures generated by Monte Carlo optimization as descriptors to predict the rat acute oral toxicity of NACs [58]. The best regression-based QSAR model showed a satisfactory validation: $R_{train}^{2}$ = 0.719, $Q_{train}^{2}$ = 0.695; $R_{test}^{2}$ = 0.739, $Q_{test}^{2}$ = 0.631. Some of the substructures are shown in Figure 1. As a result, substructures such as the presence of double-bonded oxygen, sp^2^ carbon with double bond, any hetero atom with double-bonded oxygen etc. can enhance the acute oral toxicity. Others substructures like the presence of an NH_2_ group and an sp^3^ carbon attached to the aromatic ring, the presence of sp^3^ carbon with branching, the presence of a hetero aromatic ring containing nitrogen, or the presence of oxygen and carbon etc. contribute to the decrease of NACs toxicity. More detailed substructures are listed in the original published paper [58]. Using QSAR incorporated with Monte Carlo approach significantly improved the specificity of toxicological prediction. Meanwhile, the analysis of toxicity–structure relationship towards specific substructures will further facilitate deeper understanding of the toxic mechanism.

Other than recent QSAR models based on complex quantum chemistry descriptors, Keshavarz et al., introduced models with several simple constitutional descriptors such as $n_{NO_{2}}$, $n_{S}$, $n_{P}$ (number of NO_2_, sulfur, phosphorous) and two adjustable variables *T_ox_^+^* and *T_ox_^−^* [60]. The two variables quantitatively represent a specific molecular moiety that affects the rat acute oral toxicity of NACs. Although their models were derived from simple parameters, a satisfying correlation was exhibited: $R_{adj}^{2}=0.850$, $Q_{F1}^{2}$ = 0.867, $Q_{F2}^{2}$ = 0.864, $Q_{F3}^{2}$ = 0.896. Compared with using complex quantum chemistry descriptors like other studies mentioned in this review [47,48,51,55,57,58], which usually are calculated from various softwares, the utilization of more interpretable descriptors inspires us with a new and simple approach to comprehend the structure–toxicity relationship of NACs.2.3. QSAR Studies on Mutagenicity and Carcinogenicity

### 2.3. QSAR Studies on Mutagenicity and Carcinogenicity

Mutation is a normal phenomenon in nature. From the point of view of evolution, as far as the entire biological group is concerned, the evolution of organisms and the emergence of new species are partly attributed to mutations, as well as the crossing-over process. In this case, mutations are advantageous. However, in most cases, mutations are neutral or even harmful and lethal since teratogenicity and carcinogenicity that result from mutation are detrimental. The mechanism of gene mutation is that the sequence of base pairs in DNA changes under the action of mutagen, leading to protein synthesis errors under gene control, which can be manifested as the destruction and loss of enzyme functions or structural functions. As a result, cell genetic characteristics change and subsequent mutations happen [61]. Carcinogenesis is a complex process comprised of two stages: The initiation stage is the cell gene mutation that is triggered by carcinogens; then the growth promotion stage happens, where the mutant cells change the expression of genetic information, causing the mutant cells and cancerous cells to proliferate and become tumors [62]. Generally, it is believed that carcinogenesis is closely related to mutagenesis, and most of the substances that can trigger mutations have carcinogenic effects. In this review, QSAR studies on carcinogenicity and mutagenicity of NACs are discussed in terms of different species.

#### 2.3.1. QSAR Studies on Bacteria

Among most bacteria strains, TA98 and TA100 of *S. typhimurium* strains are normally considered as the most effective candidates for mutagenicity test since they are modified to sensitively detect base-pair mutations [63]. Wang et al., built QSAR models integrated with comparative molecular field analysis (CoMFA) to predict TA98 mutagenicity of NACs [64]. In this study, classical QSAR models based on quantum chemistry descriptors, 3D-QSAR model, and 2D/3D-QSAR joint model were developed, respectively. After validation and comparison towards their predictions, 2D/3D-QSAR joint model exhibited the best performance with $R^{2}$ = 0.835 and $Q_{Loo}^{2}$ = 0.672. It is concluded that the mutagenicity of NACs incorporates molecular transportation, interaction of bio-macromolecules, and nitroreduction. Consequently, the hydrophobicity and *E_LUMO_* were considered as the indicators of nitroaromatic mutagenicity. Zhang et al., constructed QSAR models based on PLS to predict the mutagenic activity (MA) of TA98 strain of 1- and 2-nitronaphthalenes (NNs) and methylnitronaphthalenes (MNNs) [65]. The best model validation rendered a plausible outcome: *Q*^2^ = 0.711, *R* = 0.876, *p* = 1.863 × 10^−5^, *SE* = 0.53. Among 15 quantum chemistry descriptors, $∆H_{f}$, core-core repulsion energy (*CCR*), *E_HOMO_*_-1_, and *E_LUMO_* + *E_HOMO_* were demonstrated to correlate mutagenicity significantly, as shown in Equation (5). To be specific, the increase of *E_LUMO_* + *E_HOMO_* means the increase of electrophilicity, leading to more reactions with cellular nucleic acids. As for the other three parameters, the stability of mutagens decreased with the increase of their values.

(5) $$LogMA=-10.527-2.828E_{HUMO-1}-0.050∆H_{f}-0.021CCR+2.335\left(E_{LUMO}+E_{HOMO}\right)$$

In 2019, Hao et al., reported the prediction on the mutagenicity of NACs towards *Salmonella typhimurium* TA100 strain using quantum chemistry descriptor-based QSAR model and machine learning-based classification approaches [47]. They identified one quantum chemistry descriptor (*E_LUMO_*) along with four 2D descriptors (Infective-50, Hypnotic-80, CATS2D_04_LL and TIC2) that were associated to the mutagenicity of NACs, of which *E_LUMO_* and TIC2 negatively correlated with the mutagenicity, whereas the other three descriptors had a positive correlation. The key statistical parameters were *R*^2^ = 0.961, $Q_{LOO}^{2}$ = 0.950, $R_{ext}^{2}$ = 0.836, $Q_{F1}^{2}$ = 0.808, $Q_{F2}^{2}$ = 0.808, and $Q_{F3}^{2}$ = 0.826. It is worthy to note that some specific molecular properties or privileged substructures responsible for the high mutagenicity of NACs were obtained through the classification methods (substructure frequency analysis). Further, novel QSAR models combined with hierarchical support vector regression (HSVR), and PLS methods were developed by Ding et al., to predict TA98 strain mutagenicity of NACs [66]. Various descriptors were selected in different models. PLS model showed *R*^2^ = 0.72, *Q*^2^ = 0.45, *RMSE_train_* = 1.02, and *RMSE_test_* = 0.97. Apparently, this model was not ideal because several great prediction faults were observed. For HSVR models, the validation showed a satisfactory result: *R*^2^ = 0.9, *Q*^2^ = 0.84, *RMSE_train_* = 0.63 and *RMSE_test_* = 0.32, exhibiting high robustness and external predictivity. According to the prediction of QSAR models, descriptors including electrophilic index (*ω*), hydrophobicity, *E_LUMO_*, partial atomic charge on the carbon attached to the nitro group (*q_c2_*), the sum of molar refractivity of substituents at the *ortho* positions (*M_Ro_*), diNO_2_(*I*), and dipole moment can account for the complex mutagenicity of NACs—from the preliminary permeation to final base-pair mutations. Compared with previous QSAR models based on PLS, HQSAR, CoMFA, and GFA, HSVR model still outcompetes them, giving a superior stability and prediction. All these descriptors exert various mutagenic actions in different stages; further studies on mutagenic mechanism of NACs and more comprehensive QSAR models are necessary to promote the study on nitroaromatic mutagenesis.

#### 2.3.2. QSAR Studies on Mammals

Numerous QSAR studies on bacteria mutagenicity of NACs have been conducted, whereas little research on human cells has been done. Papa and co-workers constructed QSAR models to predict NPAHs mutagenicity towards human h1A1v2 cells (a line of B-lymphoblastoid cells), which were modified to express an enzyme that can metabolize PAHs [67]. Initially, 364 descriptors were selected to participate in modeling of 11 NPAHs toxicity. Unlike pure quantitative prediction, Papa et al., adopted *k*-Nearest Neighbour (*k*NN) and Classification and Regression Tree (CART) to classify prediction into two succinct groups: mutagen and non-mutagen. In this case, the validation of their QSAR models subsequently changed somehow. They used sensitivity (SE, percentage of mutagens predicted correctly), accuracy (ACC, total percentage of chemicals predicted correctly), and specificity (SP, percentage of non-mutagens predicted correctly) to quantitatively measure the performance of models. The best *k*NN model showed ACC = 81.6%, SE = 87.1%, SP = 72.2%, while CART model displayed ACC = 77.5%, SE = 87.1%, and SP = 61.1%. The two best models were chosen since they displayed the highest sensitivity in their group respectively. As a result, two vital descriptors, the 2D-topological descriptor 1-path Kier alpha-modified shape index (S1K) and the number of aromatic nitro groups (nArNO_2_) were proven to have high correlation with cellular mutagenicity.

**Figure 2:**
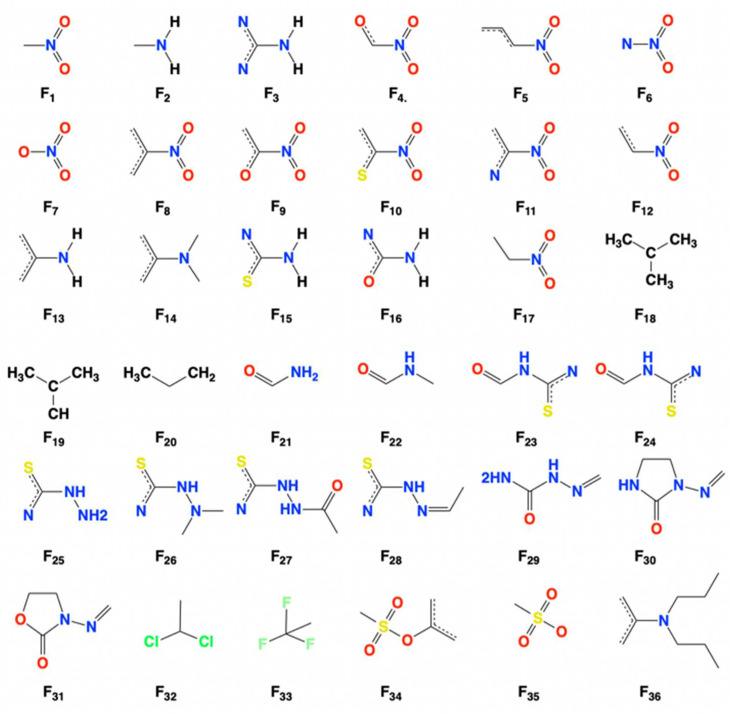
Molecular fragments contributing to carcinogenic activity of nitrocompounds.

Given the similarities of physiological structure among mammals, the toxicological investigations in rats usually promote precaution in human health. Morales et al., established QSAR models by multiple regression analysis (MRA) to predict rat carcinogenicity of nitrocompounds [68]. The carcinogenicity was quantitatively determined by TD_50_ values of female rats. After calculation of different QSAR models, the best model was generated after orthogonalization and standardization, as shown in Equation (6). These spectral moment parameters in Equation (6) refer to hydrophobicity, bond dipole moment, Gasteiger-Marsili charge, and molar refractivity. Importantly, the authors further utilized topological substructural molecular design (TOPS-MODE) to predict carcinogenicity based on the previous outcome of QSAR. Therefore, the carcinogenicity of 36 molecular fragments was described as seen in Figure 2. Table 2 quantitatively describes carcinogenicity of individual substructures. Substructures with negative values refer to the fact that they may promote carcinogenicity, while others with positive values mean the reduction of carcinogenicity.

(6) $$\log TD50=1.01+0.29\Omega^{1}\mu_{9}^{H}-0.23\Omega^{2}\mu_{6}^{H}-0.38\Omega^{3}\mu_{15}^{H}-0.22\Omega^{4}\mu_{0}\mu_{15}^{GM}\;+0.32\Omega^{5}\mu_{1}+0.16\Omega^{6}\mu_{0}-0.33\Omega^{7}\mu_{11}^{MR}\;R^{2}=0.791\;\;\;\;Q^{2}=0.666$$

Overall, the toxicity of NACs may result from various mode of interaction. One path is attacking the electro-rich position in endogenous biomolecules to exert malfunctions of protein, the other is the further reduction of hydroxylamines with individual toxicity. In addition, single-electron reduction can produce free radical anions, leading to subsequent oxidative stress and cytotoxicity after redox cycles [43]. Table 3 summarizes the recent QSAR studies on NACs toxicity in accordance with their toxic response endpoints. The mechanism of NACs toxicity in aquatic environment is not complicated compared to other toxic response endpoints. Hence, QSAR models in aquatic toxicity normally can be derived from relatively limited descriptors; even using one descriptor such as *LogK_OW_*/*LogP*/*K_OW_* is enough to get a decent prediction in some cases. For mammals, though, it usually requires more descriptors to generate satisfactory results.

## 3. Conclusions and Future Scope

In this review, QSAR studies on toxicities of NACs are classified into different genres. For aquatic toxicity, it is concluded that hydrophobicity is one of the pivotal parameters to affect the spread of NACs. This may be because the narcotic toxic effect plays a predominant role in the aquatic environment, thereby hydrophobicity determines the penetration and stimulation of toxicants towards cell membranes. Further, the toxic response endpoints of most NACs are generally close to electron-rich sites due to their electrophilic nature. Thus, *E_LUMO_* is always an indispensable indicator to correlate toxicity of NACs; the lower the *E_LUMO_* value is, the more electrophilic they are. The toxicity of NACs with more nitro groups generally depends on their electronic reactivity. That is to say, electron-withdrawing substructures like halogen will enhance toxicity. In the reductive environment, NACs are easily reduced to hydroxyl amines or other derivatives and produce free radicals, interfering with normal metabolic activities and syntheses of genetic materials in the cell. As for acute toxicity, numerous studies are conducted based on data of rodents, which further help us understand the corresponding toxicity of NACs towards human. In this case, the complexity of toxicology in mammals results in the various descriptors utilized in QSAR studies. Hydrophobicity is no longer regarded as a key factor to toxicity, instead, distinct quantum chemistry descriptors characterize toxicity to different extents. Besides, specific molecular fragments, generated from Monte Carlo or Gauss approach, are quantitatively correlated to NACs toxicity through QSAR studies, aiding us considerably in the prediction of new chemicals. Researches on the mutagenicity of NACs are imperative since genetic damages are usually irreversible. Therefore, precaution of the toxicants using QSAR models can assure environmental safety and human health. Due to the high sensitivity of TA98/100 strain to genetic mutation, they are selected as the most suitable objects to detect mutagenicity. QSAR studies reveal that dipole moment, hydrophobicity, electrophilicity, etc. contribute to mutations triggered by NACs. The QSAR studies presented above exhibit good performance, though they show some limitations in the types of test objects or applicability. Further studies in QSAR are still encouraged to establish more comprehensive and accurate models to satisfy toxicological prediction of new compounds under the regulatory framework.

## Figures and Tables

**Table 1:** Names, sources, applications, toxicity, and research objects of several representative nitroaromatic compounds.

Name	Source or Application	Toxicity	Organism	References
TNT	explosive	inhibition of cell growth and cell viability, mutagenic, liver damage, cataract	mammal	[9,10]
DNT	dye, medicine, rubber	central nervous system and respiratory system depression, ataxia, reproductive toxicity	rat	[11]
DNP	dye, pesticide, herbicide	weight loss, lethal, cataract, tumorigenicity	human	[12]
Nitrofuran	antibiotics	tumorigenicity, mutagenicity, carcinogenicity	human	[13]
Mononitrophenol	explosive, dye, pharmaceutical, rubber,	carcinogenicity	human	[14]
1-Nitropyrene	diesel	mutagenicity, carcinogenicity	human, bacteria	[15]
2-Nitronaphthalene	vehicle fuel	DNA damage	human, bacteria	[16]

**Table 2:** Molecular fragments related to carcinogenic activity.

**Studied Fragments**	Fragment Contributions	Studied Fragments	Fragment Contributions
F_1_	−0.036	F_19_	+0.188
F_2_	−0.420	F_20_	+0.649
F_3_	−0.718	F_21_	−0.235
F_4_	−0.134	F_22_	+0.581
F_5_	−0.260	F_23_	−0.466
F_6_	−0.058	F_24_	+0.791
F_7_	−0.040	F_25_	−1.390
F_8_	−0.243	F_26_	−2.329
F_9_	−0.233	F_27_	−0.799
F_10_	−0.321	F_28_	−0.779
F_11_	−0.186	F_29_	−0.661
F_12_	−0.175	F_30_	−0.202
F_13_	−1.112	F_31_	−0.092
F_14_	−0.638	F_32_	−2.770
F_15_	−0.912	F_33_	+0.010
F_16_	−0.666	F_34_	−0.871
F_17_	+0.180	F_35_	−0.605
F_18_	−1.206	F_36_	+0.890

**Table 3:** QSAR studies on the toxicity effects of NACs.

Toxicity Endpoints	Chemicals	Molecular Descriptors	Organisms	References
Aquatic Toxicity	Pesticides	PSA, $F^{+}$, $H_{f}$, S, B, V, CV	aquatic crustaceans	[41]
	Nitrophenols	Hydrophobicity, harness, electrophilicity	*Chlorella vulgaris*	[44,49]
	26 NACs	*E*_12_, BCF, *E_HOMO_*, *E_LUMO_*, *K_OW_*	*Scenedesmus obliguus*	[42]
	19 NACs	*E_HOMO_*, *E_LUMO_*, *K_OW_*, distribution coefficient	*Scenedesmus vacuolatus*	[43]
Acute Toxicity	19 NACs	*Xv*, *Kα*, Σ*σ−, E_LUMO_*, *logP* and *I*	carp	[53]
	25 NACs	*K_OW_*, *E_LUMO_* and $Q_{NO_{2}}$	*Scenedesmus obliguus*	[54]
	28 NACs	Hydrophobicity, electrostatic, Van der Waals interactions, -F and -OH	rat	[59]
	128 NACs	The van der Waals surface area, the presence of C-F at topological distance 6, and high frequency of C-N at topological distance 9	rat	[55]
	90 NACs	Presence of specific substructures generated from Monte Carlo	rat	[58]
Mutagenicity & Carcinogenicity	219 NACs	Hydrophobicity and *E_LUMO_*	TA98/100	[64]
	48 NACs	*E_HOMO_*, Hypnotic-80, Infective-50, TIC2, and CATS2D_04_LL	TA100	[47]
	16 NNs and MNNs	*H_f_*, CCR (core-core repulsion energy), *E_HOMO-1_*, *E_LUMO_* + *E_HOMO_*	TA98	[65]
	282 NACs	*ω*, hydrophobicity, *E_LUMO_*, *q_c2_*, *M*_Ro_, *I*(diNO_2_) and dipole moment	TA98	[66]
	11 NPAHs	S1K and nArNO_2_	human h1A1v2 cells	[67]
	48 NACs	Hydrophobicity, bond dipole moment, Gasteiger–Marsili charge, molar refractivity and specific molecular fragments	female rat	[68]

## Data Availability

Not applicable.
